# Should Histologic Grade Be Incorporated into the TNM Classification System for Small (T1, T2) Node-Negative Breast Adenocarcinomas?

**DOI:** 10.4061/2011/825627

**Published:** 2010-10-26

**Authors:** Mathew Purdom, Michael L. Cibull, Terry D. Stratton, Luis M. Samayoa, Edward H. Romond, Patrick C. Mcgrath, Rouzan G. Karabakhtsian

**Affiliations:** ^1^Department of Pathology & Laboratory Medicine, Chandler Medical Center, College of Medicine, University of Kentucky, 800 Rose Street, MS 129, Lexington, KY 40536, USA; ^2^Department of Behavioral Sciences, University of Kentucky, Lexington, KY 40506, USA; ^3^Comprehensive Breast Care Center, Markey Cancer Center, Lexington, KY 40536, USA

## Abstract

Prognosis of invasive ductal carcinoma (IDC) strongly correlates with tumor grade as determined by Nottingham combined histologic grade. While reporting grade as low grade/favorable (G1), intermediate grade/moderately favorable (G2), and high grade/unfavorable (G3) is recommended by American Joint Committee on Cancer (AJCC) staging system, existing TNM (Primary Tumor/Regional Lymph Nodes/Distant Metastasis) classification does not directly incorporate these data. For large tumors (T3, T4), significance of histologic grade may be clinically moot as those are nearly always candidates for adjuvant therapy. However, for small (T1, T2) node-negative (N0) tumors, grade may be clinically relevant in influencing treatment decisions, but data on outcomes are sparse and controversial. This retrospective study analyzes clinical outcome in patients with small N0 IDC on the basis of tumor grade. Our results suggest that the grade does not impact clinical outcome in T1N0 tumors. In T2N0 tumors, however, it might be prognostically significant and relevant in influencing decisions regarding the need for additional adjuvant therapy and optimal management.

## 1. Introduction

While the World Health Organization, College of American Pathologist, and American Joint Commission on Cancer all endorse reporting histologic tumor grade for IDC, it does not directly factor into the current TNM staging system [1–3]. The Nottingham Combined Histologic Grade (NCHG), the preferred grading system, stratifies tumors into three grades based on semiquantitative evaluation of tubule formation, nuclear pleomorphism, and mitoses [4]. Histologic tumor grade, as determined by NCHG, correlates with prognosis [5] and might represent a simple and inexpensive way to identify low-risk patients who are highly curable by surgery alone or are also in need of adjuvant therapy [6, 7]. Patients with large tumors are almost always candidates for adjuvant therapy, so incorporating histologic grade in such cases may be clinically irrelevant [8]. Also, tumor size often correlates with tumor grade [9]. In this era of mammographic screening, however, an increasing proportion of identified breast cancers are small and node negative. Whether or not histologic grade is an independent prognostic factor in small, node-negative IDC is still an unresolved question [10]. The Breast Task Force of the AJCC has noted that the data on this issue are sparse and inconsistent, and as such, it refrained from directly including the histologic tumor grade into the TNM staging. While the existing data clearly differentiate the prognosis of G1 and G3 tumors, the behavior of G2 tumors is ambiguous owing to methodologic differences (followup times, grading systems, and measured outcomes). We undertook a retrospective study to analyze the clinical outcomes in patients with small, node-negative cancers in an attempt to contribute to this ongoing debate regarding the prognostic significance of histologic tumor grade.

## 2. Design

The files of the Department of Pathology, University of Kentucky Medical Center were searched from January 1995 through July 2007 and yielded a total of 111 lumpectomy/mastectomy specimens from patients with T1N0 or T2N0 tumor status. The cases included 10 T1a, 23 T1b, 45 T1c, and 33 T2 tumors. The age of patients ranged from 31 to 83 years (mean, 55 years). The length of followup ranged from 7 to 152 months (mean, 56 months), with at least 60 months (5 years) and longer followup in 56% of patients. Presence of coexistent ductal carcinoma in-situ (DCIS), lymphovascular invasion (LVI), estrogen and progesterone receptor (ER/PR), and HER-2/neu status by immunohistochemistry was analyzed. Clinical followup data with outcome through year 2008 were obtained from Tumor Registry. The tumor size and histologic grade in conjunction with clinical outcome was analyzed.

## 3. Results

The data for tumor size, histologic grade, and patient status are summarized in Table 1. On the followup of patients with T1 tumors, 71/78 (93%) were alive and 3 deceased without disease. Only one patient died with disease (G1/stage T1b), and another patient was alive with disease recurrence (G2/stage T1a). Two patients were alive with status unknown. Of the patients with T2 tumors, 27/33 (82%) were alive and two deceased without disease on followup. Three died with disease (all ER negative, including one triple negative), and one was alive with disease; all four (12%) had G3 tumors (including two with LVI). All patients with hormone receptor positive tumor status received Tamoxifen or aromatase inhibitors. Of patients with T1 tumors, 24% received chemotherapy, as did 67% of patients with T2 tumors (2 of 4 with recurrent/ progressive disease had chemotherapy). 

Of T1 tumors, 76% (59/78) showed DCIS versus 69% (22/32) in T2 tumors. LVI was identified in 3% (1/33) of T1a/T1b, 13% (6/45) of T1c, and 24% (8/33) of T2 tumors. Positive ER and PR status was reported in 71% (55/78) and 64% (50/78) of T1 tumors, respectively, and 63% (20/32) and 69% (22/32) of T2 tumors, respectively. All three deceased patients with T2 tumors were tested ER negative. Two of those three tumors also showed LVI. The patient with the T1b tumor who died of disease had ER-positive tumor and no LVI. HER-2/neu status was unknown in this case. Positive HER-2/neu status was reported in 1/61 T1 tumors and 5/26 T2 tumors. Of three deceased cases, HER-2/neu status was reported in only one and was negative. Both patients who are alive with disease had positive ER and negative HER-2/neu tumors, and no LVI.

The relationship between the tumor grade and clinical outcome moderated by tumor size has been determined by using the Fisher exact tests (Table 2).

The results of the Fisher exact tests suggest an interaction effect—with the relationship between tumor grade and clinical outcome moderated by tumor size. That is, among patients with T1 tumors (*n* = 76), clinical outcome did not vary significantly by tumor grade (*P* = .46). However, among the T2 group (*n* = 33), the number of patients with G3 tumors who were with disease (*n* = 4) was significantly greater than those with G1-G2 tumors (*n* = 0)(*P* = .04). The strength of the relationships (*φ*) between tumor grade and clinical outcome for the T1 and T2 groups was.11 and.38, respectively.

## 4. Discussion

Regardless of histologic grade, the overall prognosis for small node-negative breast adenocarcinomas appears to be very good. In the current study, the disease-free survival for patients with T1N0 tumor status was 93% (71/76) and for patients with T2N0 was 88% (29/33). This relatively good prognosis is similar to that reported in prior studies [5, 11, 12], and the difficulty of addressing the prognostic significance of histologic grade in these breast cancers is highlighted. Namely, studies would need to be larger to have the statistical power to detect a relationship between histologic grade and clinical outcomes. Beyond sample size, length of followup is also an important consideration as recurrence may occur quite late [13–15]. 

 While some studies have shown histologic grade to be prognostically significant in small, node-negative breast carcinomas [5, 16, 17], other studies [18–20] do not demonstrate this association. Lundin et al. suggest that omission of histologic grading from clinical decision making may result in considerable overuse of adjuvant therapies [21]. Based on our data in this relatively small study, there is no evidence that higher tumor grade impacts the clinical outcome in T1N0 tumors. This tumor status was not associated with higher rate of recurrence or disease persistence in our study. In T2 tumors, however, our data suggest that histologic tumor grade might be prognostically significant and relevant in influencing decisions regarding the need for additional adjuvant therapy and optimal management of node-negative breast carcinomas at this stage.

 Currently, adjuvant hormonal and/or cytotoxic chemotherapies are recommended for most women with early-stage invasive breast cancer. Treatment decisions are based on axillary node status, age, tumor size, histologic tumor type, tumor grade, hormone receptor status, and coexisting medical conditions [22]. However, most patients with node-negative disease who receive chemotherapy will not derive benefit because they would not go on to have a recurrence even without such treatment, which also questions the necessity of performing the Oncotype Dx testing in T1N0 tumors. New prognostic and predictive tests are needed to better individualize therapy and confine systemic treatment, especially cytotoxic chemotherapy, to those patients who are most likely to benefit [23, 24]. Although based on a limited material, our data may suggest more favorable prognosis for patients with T1N0 regardless of tumor grade, as well as low-grade T2N0 tumors, compared to those with high-grade T2N0 disease who might benefit from additional chemotherapy. Larger studies with considerable statistical power will be needed to definitively demonstrate the impact of histologic grade in these subsets of breast adenocarcinoma.

 Several relatively recent studies indicate that the histologic tumor grade appears to reflect specific molecular predictive indicators such as proliferative markers and multigene expression arrays [25, 26]. Interestingly, the grading was shown to correlate with other proposed prognostic factors such as Recurrence Score (Oncotype Dx) and casting-type microcalcifications [23, 24, 27]. In the Kaiser population, tumor size and tumor grade remained statistically significant associated with the risk of breast cancer death in most multivariate models that also included the Recurrence Score [23], whereas only tumor grade remained independently associated with risk in the NCABP B-14 study [28]. The Recurrence Score was able to identify a larger subset of patients with low risk of breast cancer death than was possible with either of the standard prognostic indicators [23].

 While currently the Breast Cancer Task Force has elected not to include histologic grade as a stage-modifying factor in the TNM system [8], it still does recommend collection of tumor grade, using the standardized Nottingham combined histologic score with calibrated mitotic counts, for inclusion in tumor registry database [1]. How to merge histopathologic data with clinical, radiographic, and molecular information into a therapeutic plan is an evolving challenge. While many studies indicate the significance of Recurrence Score in predicting the magnitude of chemotherapy benefit, given the financial constraints and limited access to molecular testing within many health care systems, studying the utility of histologic grade (along with other parameters) continues to be relevant. For example, based on the literature as well as our data, it might appear that the Oncotype Dx testing (quoted price $4,075 per test) is adding little or no additional prognostic value to T1N0 and low-grade T2N0 tumors, which almost always show favorable outcome with no recurrence. Ultimately, determining if histologic grade will independently provide clinically relevant information in these cases to serve as a decision tool in the adjuvant chemotherapy setting merits further investigation with a large data set, extended followup, and standardized reporting.

## Figures and Tables

**Table 1:** Tumor stage, grade, and clinical outcome.

	T1a (*n* = 10)			T1b (*n* = 23)			T1c (*n* = 45)			T2 (*n* = 33)		
	G1	G2	G3	G1	G2	G3	G1	G2	G3	G1	G2	G3
Alive without disease	1	4	3	5	10	5	8	20	15	1	15	11
Alive, status unknown	—	—	1	—	1	—	—	—	—	—	—	—
Alive with disease	—	1	—	—	—	—	—	—	—	—	—	1
Deceased without disease	—	—	—	—	1	—	—	1	1	—	1	1
Deceased, cause unknown	—	—	—	—	—	—	—	—	—	—	—	—
Deceased with disease	—	—	—	1	—	—	—	—	—	—	—	3
Total	1	5	4	6	12	5	8	21	16	1	16	16

G—tumor histologic grade.

**Table 2:** Clinical outcome by tumor grade in small (T1, T2) node-negative breast adenocarcinomas.

Tumor Size	T1 (*N*=76)*					T2 (*N* = 33)				
	Clinical Outcome**					Clinical Outcome**				
	*Without Disease*		*With Disease*			*Without Disease*		*With Disease*		
Tumor Grade	*N*	%	*N*	%	P***	*N*	%	*N*	%	P***

G1-G2	50	66	2	3		17	52	0	0	
G3	24	31	0	0	.46	12	36	4	12	.04

*Excludes 2 alive, status unknown.

**Clinical outcome includes patients living and deceased.

***The *p*-value is for a one-tailed Fisher exact test.
